# A first assessment of the genetic diversity of *Mycobacterium tuberculosis *complex in Cambodia

**DOI:** 10.1186/1471-2334-11-42

**Published:** 2011-02-07

**Authors:** Jian Zhang, Seiha Heng, Stéphanie Le Moullec, Guislaine Refregier, Brigitte Gicquel, Christophe Sola, Bertrand Guillard

**Affiliations:** 1 Institut de Génétique et Microbiologie, UMR8621 CNRS-Université Paris-Sud 11, UniverSud, Infection Genetics Emerging Pathogens Evolution (IGEPE) Team, Bât. 400, F-91405 Orsay-Cedex, France; 2 Institut Pasteur du Cambodge, Phnom-Penh, 5 boulevard Monivong, BP 983, Phnom-Penh, Royaume du Cambodge; 3 Unité de Génétique Mycobactérienne, Institut Pasteur, Paris, France

## Abstract

**Background:**

Cambodia is among the 22 high-burden TB countries, and has one of the highest rates of TB in South-East Asia. This study aimed to describe the genetic diversity among clinical *Mycobacterium tuberculosis *complex (MTC) isolates collected in Cambodia and to relate these findings to genetic diversity data from neighboring countries.

**Methods:**

We characterized by 24 VNTR loci genotyping and spoligotyping 105 *Mycobacterium tuberculosis *clinical isolates collected between 2007 and 2008 in the region of Phnom-Penh, Cambodia, enriched in multidrug-resistant (MDR) isolates (n = 33).

**Results:**

Classical spoligotyping confirmed that the East-African Indian (EAI) lineage is highly prevalent in this area (60%-68% respectively in whole sample and among non-MDR isolates). Beijing lineage is also largely represented (30% in whole sample, 21% among non-MDR isolates, OR = 4.51, CI_95% _[1.77, 11.51]) whereas CAS lineage was absent. The 24 loci MIRU-VNTR typing scheme distinguished 90 patterns with only 13 multi-isolates clusters covering 28 isolates. The clustering of EAI strains could be achieved with only 8 VNTR combined with spoligotyping, which could serve as a performing, easy and cheap genotyping standard for this family. Extended spoligotyping suggested relatedness of some unclassified "T1 ancestors" or "Manu" isolates with modern strains and provided finer resolution.

**Conclusions:**

The genetic diversity of MTC in Cambodia is driven by the EAI and the Beijing families. We validate the usefulness of the extended spoligotyping format in combination with 8 VNTR for EAI isolates in this region.

## Background

Tuberculosis (TB) as caused by *Mycobacterium tuberculosis *complex (MTC) is one of the most important public health problems in the world. In 2008, WHO estimated 9.4 million incident cases of TB, 11.1 million prevalent cases, 1.3 million deaths among HIV-negative people and an additional 0.52 million TB deaths among HIV-positive people [1]. The largest number of new TB cases occurs in the South-East Asian Region, that accounted for 34% of incident cases in 2006 [2].

Cambodia is among the 22 high-burden TB countries, and has one of the highest rates of TB in South-East Asia [3]. The population of Cambodia is 13.4 million (Population Census of 2008). The new sputum smear positivity rate for Cambodia is estimated to be 220/100,000 inhabitants whereas the TB incidence is estimated around 500/100,000 inhabitants per year and the mortality rate at 94/100,000 inhabitants [4]. Phnom-Penh is the capital of Cambodia, with more than 1.3 million inhabitants (Population Census of 2008), which represents 10% of the whole population living in the country. The population density of Phnom-Penh is the highest of the country, with around 4,500 people per km^2^.

Except for studies done in neighboring countries (Vietnam, Thailand and Myanmar) nothing is known about the genetic diversity of MTC in Cambodia [5-7]. The goal of this study was to describe the genetic diversity of MTC in Cambodia.

Multidrug resistant TB (MDR-TB) is an important aspect of tuberculosis control [8,9]. A high level of resistance to Isoniazid (Inh) and Rifampin (Rif) was identified in Phnom Penh in patients co-infected with HIV, and the minimal number of MDR-TB identified new cases in 2009 in Cambodia was 94 [4].

We focused on a restricted population sample (n = 118), mixing randomly drawn (n = 59) and enriched in MDR-TB clinical isolates (n = 59) according to drug susceptibility testing results. We report in this article the characterization of this 118 clinical isolates collection as a first insight on the genetic population structure of MTC in Cambodia.

## Results

### Spoligotyping, classical 43 spacers format

*M. tuberculosis *complex clinical isolates were collected from 118 patients. DNA samples were extracted from subcultures by a thermolysis procedure. Patient demographic characteristics were as follows: a median age of 38 years, and a sex ratio close to 1 (Table 1). We obtained a 43-spacers spoligotyping result for 113 DNA samples. A total of 42 individual patterns were found, among which, 27 patterns were unique whereas 85 clinical isolates were distributed in 15 clusters containing 2 to 31 isolates (Table 2). Eighteen patterns were not previously reported in the SpolDB4 international database fifteen of which presenting the typical signature of the East African-Indian (EAI) family (absence of spacers 29-32, presence of spacer 33, absence of spacer 34) [10]. Two other previously unreported isolates (isolates Cam108 and Cam116) carried a T1-ancestor or "Manu" signature (all spacers present except 33 and/or 34) and one isolate harbored a very unusual pattern (spacer 1-3 only were present) [11]. Altogether, EAI is clearly the predominant genetic family in Cambodia. It totaled 67 clinical isolates (59% of successfully genotyped isolates) but up to 56 of the 84 successfully typed non-MDR (67%). The Beijing family encompassed 34 isolates, (30% of the total successfully genotyped isolates) but only 18 of non-MDR typed isolates (21%). In Beijing lineage, 26 out of 34 (76%) isolates were resistant to at least one of the drug (isoniazid, rifampin, streptomycin, ethambutol) while only 34% were among EAI isolates. The weight of these two lineages significantly differed between non-MDR and MDR isolates (OR = 4.52; CI_95% _[1.78,11.51]). Other minor families were: the T (Modern) family (with a total of 7 clinical isolates or 6%), some U (unclassified with likely "T1-ancestor" or "Manu" signatures) patterns (4 clinical isolates or 3.5%) and 3 other undefined isolates, all together representing only ∼10% of total isolates.

**Table 1:** Characteristics of the 118 patients

Age, y	
Median (range)	38 (9-77)
Sex, n° (%)	
Male	58 (50.9)
Female	56 (49.1)

Consultation in hospital in, n° (%)	
Phnom Penh	81 (68.6)
Province	37 (31.4)
Multidrug resistant strains, n° (%)	33 (28.0)

**Table 2:** Discriminatory powers of the different genotyping methods, for the whole sample and for the two major families

Method	Strains	Types	Clusters	Cluster size	HGDI*	HGDI EAI	HGDI Beijing
MIRU-VNTR 24 loci	105	92	13	2-4	0,997	0,994	0,991
spoligotyping 68 spacers	113	51	15	2-29	0,925	0,963	0,229
spoligotyping 43 spacers	113	42	15	2-31	0,906	0,932	0,119

HGI = Hunter and Gaston Discriminatory Index

### Spoligotyping, extended 68 spacers format

**Figure 1:**
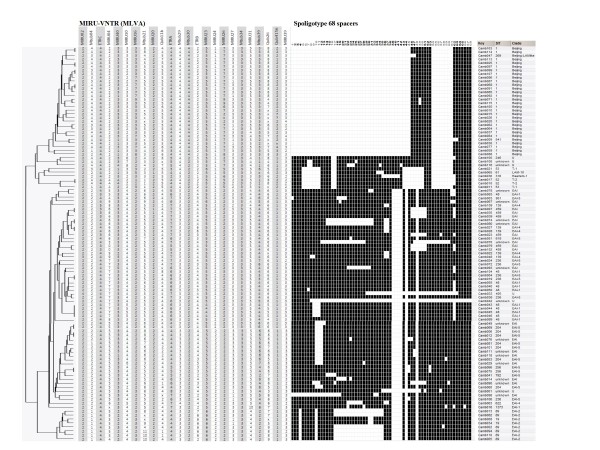
**Genotyping results and dendrogram of Cambodian *Mycobacterium tuberculosis *clinical isolates**. From left to right: 1) UPGMA dendrogram of the 105 genotypes obtained on 24 MIRU-VNTR loci (*M. tuberculosis *MLVA standard). 2) Number of repetitions of each VNTR according to Supply et al. nomenclature. 3) 68-spacers Spoligotypes: black spots represent the presence and white spots represent the absence of 1-68 spacer (according to J. van Embden *et al. *numbering [27]. 4) Strain number (internal nomenclature). 5) SIT = Spoligo-International-Type. 6) Genetic family (clade) according to SpolDB4 [38].

The use of 25 additional spacers significantly improved the discriminatory power. 51 patterns (+21%) were distinguished among which 36 were unique patterns. 77 isolates were distributed in a total of 15 clusters (Table 2). In the Beijing family (Spoligotyping-International-Type, SIT1), 2 subtypes were observed, among which, one cluster of only 2 isolates. Within the EAI family, several subtypes were observed by 68 spacers' spoligotyping (see Additional file 1 and Figure 1). Among the predominant clusters, two SIT signatures (SIT459, SIT204) were found to be prevalent and each split into two subtypes. Genotyping with the 25 extra spacers revealed that the "T1-ancestor" or "Manu" isolates all harbored the same signature (absence of spacers 54-61) as do isolates from Principal Genetic Groups 2 and 3 modern isolates. The common ancestry of these "T1 ancestor" or "Manu" isolates with the "Latin-American and Mediterranean (LAM), the "Haarlem" (H), the "Anglo-Saxon" (X), other less-defined "T-related" and some U (unknown) isolates is further supported by their clustering based on MLVA genotyping (see below and Figure 1).

### MIRU-VNTR (Multiple Locus VNTR or MLVA) Typing

Hundred and five (105) clinical isolates out of 118 gave interpretable results. QuB26 failure rate was the highest (no amplification for 10 isolates); for three isolates, we had no amplification of Qub4156 and for three others we had no amplification of Mtub21. For some isolates a single VNTR was missing. The 24 loci internationally-agreed MIRU-VNTR typing scheme allowed to distinguish 13 clusters totaling 28 isolates (Table 2). Three isolates (Cam001, Cam033 and Cam042) which were unclassified by spoligotyping were similar to EAI isolates by MIRU-VNTR. In particular, isolate Cam033 (SIT405) differed by a single locus variation (SLV in MIRU02) from isolate Cam038 which belongs to the EAI2_Manilla subfamily. The discriminatory powers of each MIRU-VNTR locus respectively for the whole sample and for EAI or Beijing lineage were estimated by calculating the Hunter and Gaston Discriminatory Index (HGDI) (Table 3). Nine loci included in the standardized 24 VNTR scheme were uninformative to genotype Beijing clinical isolates. For Beijing isolates, the most discriminatory loci were (in a decreasing order): QuB26, MIRU10, MIRU16, Mtub04, Mtub21 and QuB11b. Using these 6 loci, only 2 strains remained clustered. For the EAI isolates of MTC, the most discriminatory loci were (in a decreasing order): ETRD, Mtub39, ETRA, Mtub21, ETRB and QuB26.

**Table 3:** Hunter Gaston Discriminatory Index (HGDI) of each locus of Multiple Loci VNTR Analysis (MLVA) for the whole sample and for the two major families

	ETRA	ETRB	ETRC	ETRD	ETRE	MIRU02	MIRU10	MIRU16	MIRU20	MIRU23	MIRU24	MIRU26
Total	0,67	0,68	0,14	0,79	0,40	0,04	0,62	0,57	0,06	0,27	0,49	0,52
EAI	0,73	0,56	0,23	0,73	0,38	0,03	0,24	0,18	0,06	0,38	0,03	0,03
Beijing	0,07	0,00	0,00	0,13	0,13	0,00	0,51	0,50	0,00	0,00	0,00	0,37

	MIRU27	MIRU39	MIRU40	Mtub04	Mtub21	Mtub29	Mtub30	Mtub34	Mtub39	Qub11b	Qub26	Qub4156

Total	0,27	0,24	0,38	0,60	0,65	0,47	0,41	0,02	0,77	0,62	0,69	0,45
EAI	0,18	0,12	0,30	0,25	0,62	0,03	0,00	0,00	0,66	0,30	0,53	0,03
Beijing	0,33	0,30	0,34	0,47	0,45	0,00	0,00	0,00	0,13	0,40	0,59	0,28

### Combined Spoligotyping (68 spacers) and MIRU-VNTR analysis

The number of clusters (defined as 100% identical isolates on both 24 VNTR and spoligotyping) was low (n = 12) and represented a total of 26 clinical isolates (Figure 1). Among these clusters, a single cluster of 4 clinical isolates (SIT48) was observed, whereas all others were micro-clusters of 2 clinical isolates. Interestingly, two of these microclusters were formed of MDR Beijing isolates.

The most prevalent Spoligo-International-Types (SIT) were SIT204 and SIT459, both belonging to the EAI family. They were searched for in the genetic databases SITVIT2 http://www.pasteur-guadeloupe.fr and MIRU-VNTRplus to look for the geographical distribution of these two types [12,13]. The search within SITVIT2 of SIT204 (737777777413771), retrieved six clinical isolates among which two were from patients living in neighboring Vietnam and Thailand. No similar isolates were found in MIRU-VNTR plus database. The search within SITVIT2 of SIT459 (777777777410331) retrieved three clinical isolates (from France and USA) without more information on the origin of patients. In MIRU-VNTR plus database, a 4 loci strain variant (12778/03) was found for this SIT. This MIRU-VNTR plus database isolate harbored SIT152, which differs by a single spacer from SIT139, the prototypic spoligotype from the "Hanoi strain" (also designated as EAI4_Vietnam) [10]. This confirms the relatedness to EAI lineage of the SIT459 isolates included in our study.

By using the 68 spacer spoligotyping scheme and simultaneously the 6 most discriminant loci for EAI isolates (ETRD, Mtub39, ETRA, Mtub21, ETRB and QuB26), we obtained almost the same clustering result as by using the 24 VNTR loci. Only two clusters of two EAI isolates were not distinguished using this combined scheme: isolates Cam111 and Cam118 that differ by MIRU40 only, and isolates Cam79 and Cam102 that differ by QuB11b only. Considering that isolates that differ by a single marker using MLVA-24 have an equal likelihood to represent a non epidemiologically-informative cluster as an epidemiologically one (due to genetic convergence on MIRU-VNTR), we suggest that these 6 VNTR loci combined to spoligotyping scheme could be sufficient to genotype EAI strains in this part of the world for epidemiological purposes. Including two additional markers (MIRU40 and QuB11b), i.e. 8 VNTR loci could further strengthen the clustering results.

## Discussion

This study provides for the first time an insight on the genetic diversity of *Mycobacterium tuberculosis *complex in Cambodia. Although preliminary because our sampling was a mixed susceptible, mono-resistant, and MDR clinical isolates and did not cover a sufficient period of time and sufficient clinical isolates number, we show that the population structure of *M. tuberculosis *in Cambodia is dominated by the EAI and Beijing families and is depleted of the Central Asian (CAS) family. Indeed, these two families represent almost 90% of the total population (59-67% for EAI and 21-30% for Beijing depending whether the full set or the susceptible set only is taken to be representative of the actual diversity). Other modern families like T, Haarlem and Latin-American and Mediterranean (LAM) represent anecdotal cases.

Compared to previous studies performed in the South and South-East Asian region (India, Bangladesh, Myanmar, Thailand, Vietnam and this study) we observe that the CAS lineage is totally absent in Cambodia and Vietnam only [6,7,9,14-16]. Indonesia and Malaysia seem to be the lower south-eastern borders of the CAS lineage distribution in South-East Asia. Conversely an increased gradient of the EAI lineage from to India to South-East Asia is observed (Bangladesh: EAI, 27%; CAS, 16%, Beijing, 33%; Myanmar: EAI, 48%; CAS, 5%, Beijing 32%; Vietnam: EAI, 51%, Beijing, 32%; Cambodia: EAI, 60%, Beijing 30%). In India, where the relative CAS/EAI distribution remains to be studied with more accuracy in relation to human populations, it was shown that the CAS lineage is predominant in the North (56%) and sporadic in the South (1%) while the EAI lineage proportion increases from 27% in the North to 89% in the South [17].

The MDR-TB clinical isolates were shown to belong to three lineages: Beijing, EAI and "T1-ancestor" also designated as "Manu". The distribution of non-MDR and MDR-TB clinical isolates within the lineages was significantly different: whereas Beijing strains represented only 21% of non-MDR isolates, this percentage increased to around 50% among MDR-TB clinical isolates. We also observed two Beijing clusters which contained two perfectly identical MDR-clinical isolates each, whereas no such cases were found among EAI MDR strains. With the available data, the hypothesis of MDR-TB transmission could not be rejected. Drug-resistance thus could be associated with Beijing family as already observed in Vietnam, Thailand and South Africa [6,9,18-22]. The exact features of MDR-TB in this region clearly warrant further studies.

Another goal of this study was also to investigate the discriminatory power of alternative future genotyping schemes. Although the 24 loci MIRU-VNTR typing (MLVA) is actually the most powerful genotyping method for *M. tuberculosis*, it has some technical limitations: in lower resource countries, MIRU-VNTR can be only performed manually. As these countries have often a high TB prevalence, the work can be very time-consuming and tedious. 24 VNTR typing may also be run on capillary electrophoresis thus achieving higher efficiency. Spoligotyping is a first-line rapid genotyping method, even when performed on membrane since it can analyze up to 40 samples per experiment in one day. When combined with few highly discriminatory VNTR loci, spoligotyping can achieve a discriminatory power similar to that obtained with the 24-VNTR scheme, with much less working load and time. In this study, a combination of spoligotyping with 8 VNTR loci (ETRD, Mtub39, ETRA, Mtub21, ETRB, QuB26, MIRU40 and QuB11b) allows to obtain the same discriminatory power than the MIRU-VNTR 24 loci-scheme for EAI strains (66 isolates). Thus, typing scheme in Myanmar, Bangladesh, Laos and Cambodia could consist in spoligotyping to identify main families, followed by a restricted 8-VNTR panel for EAI isolates, while the 24 VNTR panel would remain mandatory for the remaining MTC clinical isolates.

## Conclusions

This study describes TB genetic diversity in Cambodia and preliminary information on drug-resistance features in this country. Our results support the absence of CAS family such as in Vietnam.

## Methods

### Patient Characteristics

Samples were collected from January 2007 to October 2008 from inpatients and outpatients consulting in Phnom Penh and in provincial hospitals. Strains were obtained from fresh material: sputum (n = 93), broncho-aspirates (n = 5), stool (n = 5), gastric aspirates (n = 3), lymph node aspirates (n = 3), cerebrospinal fluid (n = 2), urine (n = 1), blood culture (n = 1) and other (n = 5). The characteristics of the 118 patients are shown in Table 1. The mean age of the patients is 37.66 years. 58 men and 56 women, the male to female ratio equal to 1.03, slightly higher than the sex ratio of the region of Phnom-Penh (0.9) (no information for 4 patients).

#### Mycobacterium tuberculosis isolation and identification, drug susceptibility patterns

No formal informed consent was obtained for this study since the study involved clinical isolates obtained during routine diagnostic work. Sputum decontamination was performed by the sodium-lauryl-sulfate method with direct examination by the auramine technique followed by culture on Löwenstein-Jensen and classical identification using the niacin test [23]. First-line drug susceptibility testing was done in BBL MGIT tubes (Becton Dickinson, New Jersey, USA) with appropriate fluorescence detection using the manual technique.

### Clinical isolates and Drug Susceptibility testing patterns

118 clinical isolates of *M. tuberculosis *from the collection of Institut Pasteur of Cambodia were selected for this study. All samples were isolated during 2007 to 2008 in the region of the capital city Phnom-Penh (n = 81) as well as from Kampong Cham (n = 25), Takéo (n = 8), and others (n = 4, Siem Reap and Sihanouk Ville). The whole sample results from the addition of one totally random (n = 59) from 2007 and one collection enriched in MDR strains from both 2007 and 2008 (n = 59, n_MDR _= 31) as evidenced by their drug-susceptibility testing profiles. The whole sample contained 59 strains (50%) which were susceptible for all 4 drugs tested (Rifampin or Rif, Isoniazid or Inh, Streptomycin or Srm and Ethambutol or Emb). 47 clinical isolates were resistant to isoniazid, 43 were resistant to streptomycin, 34 were resistant to rifampin, and 14 resistant to ethambutol. 33 clinical isolates were MDR strains (28%), 12 of which isolated during 2007 and 21 isolated during 2008. 12 clinical isolates were resistant to all tested four first line drugs.

### DNA extraction method

MTC Colonies were scraped from LJ slopes, collected into Eppendorf tubes containing Tris-EDTA buffer (10 mM-1 mM, pH 8.0), heated for 30 min at 95°C and freeze in ethanol-dry ice bath. After centrifugation at 13000 g, the resulting aqueous phase was transfered to new tubes and kept at -20°C until further use.

### Genotyping Methods

DNAs were sent by express carrier to the Institut Pasteur and an aliquot was transferred to the Institut of Genetics and Microbiology to be genotyped. Both spoligotyping and MIRU-VNTR were used as standard genotyping procedures.

Spoligotyping: The PCR was performed according to the described protocol with a total of 20 cycles [24]. Spoligotyping was performed by using the microbeads-based platform Luminex^® ^200 [25,26]. In addition to the classical 43 spacers' spoligotyping, an extended spoligotyping assay with 68 spacers, using 25 extra spacers was performed [27,28]. Family assignations were performed by searching in SpolDB4 database [10]. The corresponding naming was used. For a reminder, EAI is sometimes called "Indo-Oceanic"; Beijing, "East-Asian"; CAS, "East-African-Indian"; and modern Principal Genetic Group 2 and 3 strains, "Euro-American lineage" [29].

MIRU-VNTR typing (also called Multi-Locus VNTR analysis or MLVA) was done as described previously using the standardized 24-loci VNTR typing scheme [30]. Loci analyzed were as follows; the 5 ETRs (exact tandem repeats), ETRA, B, C, D, E [31]. The 12 MIRUs (Mycobacterial Interspersed Repetitive Unit), MIRU-2, 4, 10, 16, 20, 23, 26, 27, 31, 37, 39, 40 (except for the redundant ETR-D and ETR-E, *i.e. *MIRU- 4 and MIRU -31 which were not retyped) [32,33]. The 6 Mtubs: Mtub-4, 21, 29, 30, 34, 39 and the 3 QuBs (Queen University Belfast) QuB-11b, 4156, 26 [34,35]. The length of the PCR products and number of repeats were estimated by eye after electrophoresis using agarose Gels 2% and a corresponding table concordant with that of MIRU-VNTR+ website [12].

### Statistical and clustering analysis

The discriminatory powers of different typing methods were estimated by computing the numerical index of discrimination, the Hunter-Gaston Discriminatory Index (HGDI), as described previously [36]. Markers with missing data were not included for the calculation of the HGDI. The Recent Transmission Index (RTI) was computed using the (n-1) method [37]. Dendrogram was drawn using the UPGMA (Unweighted Pair Group Method using Arithmetic averages) implemented in Bionumerics 5.1 version software (Applied Maths, Sint-Marten-Latems, Belgium) following the methods described in user's manual and with missing data coded as "0".

To compare the proportion of each lineage among MDR and non-MDR strains, odds ratio were computed in Excel^® ^spreadsheet files.

## Competing interests

The authors declare that they have no competing interests.

## Authors' contributions

BG, BG and CS participated to the design of the study. SH participated to culture and isolation of MTC diagnostics as well as DNA extraction and was trained to spoligotyping in Paris; JZ and SLM respectively performed spoligotyping and VNTR, JZ participated to data analysis with GR and CS. JZ, GR and CS participated to the drafting of the manuscript. B. Gicquel and CS participated to the supervision of the study and critical revision of the manuscript. All authors read and approved the final version of the manuscript.

## Pre-publication history

The pre-publication history for this paper can be accessed here:

http://www.biomedcentral.com/1471-2334/11/42/prepub

## Supplementary Material

Additional file 1 **43-spacers spoligotypes subdivised by extended 68-spacers spoligotyping.** cf. Figure 1 for full description of all 68-spacerssubtypes Click here for file

## References

[B1] WHO Global Tuberculosis Control: Surveillance, Planning, Financing 2008 WHO, Geneva, Switzerland

[B2] WHO Global Tuberculosis Control: Surveillance, Planning, Financing 2006 WHO, Geneva, Switzerland

[B3] USAID Infectious Diseases http://www.usaid.gov/our_work/global_health/id/tuberculosis/countries/asia/cambodia_profile.html accessed January 29 ^th ^2011

[B4] Sar B Keo C Leng C Saman M Min DC Chan S Monchy D Sarthou JL Anti-tuberculosis drug resistance and HIV co-infection in Phnom Penh, Cambodia Southeast Asian J Trop Med Public Health 2009 40 1 104 107 19323041

[B5] Smittipat N Billamas P Palittapongarnpim M Thong-On A Temu MM Thanakijcharoen P Karnkawinpong O Palittapongarnpim P Polymorphism of variable-number tandem repeats at multiple loci in *Mycobacterium tuberculosis* J Clin Microbiol 2005 43 10 5034 5043 10.1128/JCM.43.10.5034-5043.2005 16207958 PMC1248453

[B6] Buu TN Huyen MN Lan NT Quy HT Hen NV Zignol M Borgdorff MW Cobelens FG van Soolingen D The Beijing genotype is associated with young age and multidrug-resistant tuberculosis in rural Vietnam Int J Tuberc Lung Dis 2009 13 7 900 906 19555542

[B7] Phyu S Jureen R Ti T Dahle UR Grewal HM Heterogeneity of *Mycobacterium tuberculosis *isolates in Yangon, Myanmar J Clin Microbiol 2003 41 10 4907 4908 10.1128/JCM.41.10.4907-4908.2003 14532259 PMC254380

[B8] Wells CD Cegielski JP Nelson LJ Laserson KF Holtz TH Finlay A Castro KG Weyer K HIV infection and multidrug-resistant tuberculosis: the perfect storm J Infect Dis 2007 196 Suppl 1 S86 107 10.1086/518665 17624830

[B9] Cheunoy W Haile M Chaiprasert A Prammananan T Cristea-Fernstrom M Vondracek M Chryssanthou E Hoffner S Petrini B Drug resistance and genotypic analysis of *Mycobacterium tuberculosis *strains from Thai tuberculosis patients Apmis 2009 117 4 286 290 10.1111/j.1600-0463.2009.02438.x 19343824

[B10] Brudey K Driscoll J Rigouts L Prodinger WM Gori A Al-Hajoj SAM Allix C Aristimuno L Arora J Baumanis V *Mycobacterium tuberculosis *complex genetic diversity: mining the fourth international spoligotyping database (SpolDB4) for classification, Population Genetics, and Epidemiology BMC Microbiol 2006 6 6 23 10.1186/1471-2180-6-23 16519816 PMC1468417

[B11] Singh UB Suresh N Vijaya Bhanu N Arora J Pant H Sinha S Aggarwal RC Singh S Pande JN Sola C Predominant Tuberculosis Spoligotypes, Delhi, India Emerg Infect Dis 2004 10 6 1138 1142 15207071 10.3201/eid1006.030575 PMC3323169

[B12] Allix-Beguec C Harmsen D Weniger T Supply P Niemann S Evaluation and user-strategy of MIRU-VNTRplus, a multifunctional database for on-line analysis of genotyping data and phylogenetic identification of *Mycobacterium tuberculosis *complex isolates J Clin Microbiol 2008 46 8 2692 9 Epub 2008 Jun 11 10.1128/JCM.00540-08 18550737 PMC2519508

[B13] Helal ZH Ashour MS Eissa SA Abd-Elatef G Zozio T Babapoor S Rastogi N Khan MI Unexpectedly high proportion of ancestral Manu genotype *Mycobacterium tuberculosis *strains cultured from tuberculosis patients in Egypt J Clin Microbiol 2009 47 9 2794 2801 10.1128/JCM.00360-09 19553569 PMC2738058

[B14] Bhanu NV van Soolingen D van Embden JD Dar L Pandey RM Seth P Predominance of a novel Mycobacterium tuberculosis genotype in the Delhi region of India Tuberculosis (Edinb) 2002 82 2-3 105 112 10.1054/tube.2002.0332 12356462

[B15] Banu S Gordon SV Palmer S Islam R Ahmed S Alam KM Cole ST Brosch R Genotypic Analysis of *Mycobacterium tuberculosis *in Bangladesh and Prevalence of the Beijing Strain J Clin Microbiol 2004 42 2 674 682 10.1128/JCM.42.2.674-682.2004 14766836 PMC344461

[B16] Rahim Z Zaman K van der Zanden AG Mollers MJ van Soolingen D Raqib R Zaman K Begum V Rigouts L Portaels F Assessment of population structure and major circulating phylogeographical clades of *Mycobacterium tuberculosis *complex in Bangladesh suggests a high prevalence of a specific subclade of ancient *M. tuberculosis *genotypes J Clin Microbiol 2007 45 11 3791 3794 10.1128/JCM.01247-07 17804653 PMC2168514

[B17] Gutierrez MC Ahmed N Willery E Narayanan S Hasnain SE Chauhan DS Katoch VM Vincent V Locht C Supply P Predominance of ancestral lineages of *Mycobacterium tuberculosis *in India Emerg Infect Dis 2006 12 9 1367 1374 17073085 10.3201/eid1209.050017 PMC3294724

[B18] Johnson R Warren R Strauss O Jordaan A Falmer A Beyers N Schaaf H Murray M Cloete K van Helden P An outbreak of drug-resistant tuberculosis caused by a Beijing strain in the western Cape, South Africa Int J Tuberc Lung Dis 2006 10 12 1412 1414 17167961

[B19] Glynn JR Whiteley J Bifani PJ Kremer K Van Soolingen D Worldwide Occurrence of Beijing/W Strains of *Mycobacterium tuberculosis*: A Systematic Review Emerg Infect Dis 2002 8 8 843 849 12141971 10.3201/eid0808.020002 PMC2732522

[B20] Lan NTN Lien HTK Tung LB Borgdorff MW Kremer K Van Soolingen D *Mycobacterium tuberculosis *Beijing genotype and risk for treatment failure and relapse, Vietnam Emerg Infect Dis 2003 9 1633 1635 14720411 10.3201/eid0912.030169 PMC3034339

[B21] Anh DD Borgdorff MW Van LN Lan NT van Gorkom T Kremer K van Soolingen D *Mycobacterium tuberculosis *Beijing genotype emerging in Vietnam Emerg Infect Dis 2000 6 3 302 305 10.3201/eid0603.000312 10827122 PMC2640863

[B22] Prammananan T Cheunoy W Taechamahapun D Yorsangsukkamol J Phunpruch S Phdarat P Leechawengwong M Chaiprasert A Distribution of *rpoB *mutations among multidrug-resistant *Mycobacterium tuberculosis *(MDRTB) strains from Thailand and development of a rapid method for mutation detection Clin Microbiol Infect 2008 14 5 446 453 10.1111/j.1469-0691.2008.01951.x 18294243

[B23] David H Levy-Frebault V Thorel MF Méthodes de laboratoire pour Mycobactériologie clinique 1989 Paris: Institut Pasteur 1 87

[B24] Kamerbeek J Schouls L Kolk A van Agterveld M van Soolingen D Kuijper S Bunschoten A Molhuizen H Shaw R Goyal M Simultaneous detection and strain differentiation of Mycobacterium tuberculosis for diagnosis and epidemiology J Clin Microbiol 1997 35 4 907 914 9157152 10.1128/jcm.35.4.907-914.1997 PMC229700

[B25] Cowan LS Diem L Brake MC Crawford JT Transfer of a *Mycobacterium tuberculosis *genotyping method, Spoligotyping, from a reverse line-blot hybridization, membrane-based assay to the Luminex multianalyte profiling system J Clin Microbiol 2004 42 1 474 477 10.1128/JCM.42.1.474-477.2004 14715809 PMC321738

[B26] Zhang J Abadia E Refregier G Tafaj S Boschiroli ML Guillard B Andremont A Ruimy R Sola C *Mycobacterium tuberculosis *complex CRISPR genotyping: improving efficiency throughput and discriminative power of 'spoligotyping' with new spacers and a microbead-based hybridization assay J Med Microbiol 2009 59 285 294 10.1099/jmm.0.016949-0 19959631

[B27] van Embden JDA van Gorkom T Kremer K Jansen R van der Zeijst BAM Schouls LM Genetic variation and evolutionary origin of the Direct repeat locus of *Mycobacterium tuberculosis *complex bacteria J Bacteriol 2000 182 2393 2401 10.1128/JB.182.9.2393-2401.2000 10762237 PMC111299

[B28] Brudey K Gutierrez MC Vincent V Parsons LM Salfinger M Rastogi N Sola C *Mycobacterium africanum *Genotyping Using Novel Spacer Oligonucleotides in the Direct Repeat Locus J Clin Microbiol 2004 42 11 5053 5057 10.1128/JCM.42.11.5053-5057.2004 15528695 PMC525283

[B29] Gagneux S DeRiemer K Van T Kato-Maeda M de Jong BC Narayanan S Nicol M Niemann S Kremer K Gutierrez MC Variable host-pathogen compatibility in *Mycobacterium tuberculosis* Proc Natl Acad Sci USA 2006 103 8 2869 2873 10.1073/pnas.0511240103 16477032 PMC1413851

[B30] Supply P Allix C Lesjean S Cardoso-Oelemann M Rusch-Gerdes S Willery E Savine E de Haas P van Deutekom H Roring S Proposal for Standardization of Optimized Mycobacterial Interspersed Repetitive Unit-Variable-Number Tandem Repeat Typing of *Mycobacterium tuberculosis* J Clin Microbiol 2006 44 12 4498 4510 10.1128/JCM.01392-06 17005759 PMC1698431

[B31] Frothingham R Meeker-O'Connell WA Genetic diversity in the *Mycobacterium tuberculosis *complex based on variable numbers of tandem DNA repeats Microbiology 1998 144 Pt 5 1189 1196 10.1099/00221287-144-5-1189 9611793

[B32] Supply P Mazars E Lesjean S Vincent V Gicquel B Locht C Variable human minisatellite-like regions in the *Mycobacterium tuberculosis *genome Mol Microbiol 2000 36 762 771 10.1046/j.1365-2958.2000.01905.x 10844663

[B33] Supply P Lesjean S Savine E Kremer K van Soolingen D Locht C Automated high-throughput genotyping for study of global epidemiology of *Mycobacterium tuberculosis *based on mycobacterial interspersed repetitive units J Clin Microbiol 2001 39 3563 3571 10.1128/JCM.39.10.3563-3571.2001 11574573 PMC88389

[B34] Le Fleche P Fabre M Denoeud F Koeck JL Vergnaud G High resolution, on-line identification of strains from the *Mycobacterium tuberculosis *complex based on tandem repeat typing BMC Microbiol 2002 2 1 37 10.1186/1471-2180-2-37 12456266 PMC140014

[B35] Skuce RA McCorry TP McCarroll JF Roring SM Scott AN Brittain D Hughes SL Hewinson RG Neill SD Discrimination of *Mycobacterium tuberculosis *complex bacteria using novel VNTR-PCR targets Microbiology 2002 148 Pt 2 519 528 11832515 10.1099/00221287-148-2-519

[B36] Hunter PR Gaston MA Numerical index of the discriminatory ability of typing systems: an application of Simpson's index of diversity J ClinMicrobiol 1988 26 2465 2466 10.1128/jcm.26.11.2465-2466.1988 PMC266921 3069867

[B37] Small PM Hopewell PC Singh SP Paz A Parsonnet J Ruston DC Schecter GF Daley CL Schoolnik GK The Epidemiology of Tuberculosis in San Francisco N Engl J Med 1994 330 24 1703 1709 10.1056/NEJM199406163302402 7910661

[B38] SpolDB4 http://www.pasteur-guadeloupe.fr/tb/bd_myco.html

