# Supplementary material for: A first assessment of the genetic diversity of Mycobacterium tuberculosis complex in Cambodia
Source: BMC Infect Dis. 2011 Feb 7;11:42. doi: 10.1186/1471-2334-11-42 (PMC3062598; doi:10.1186/1471-2334-11-42)
Supplement: Additional file 1 — 43-spacers spoligotypes subdivised by extended 68-spacers spoligotyping. cf. Figure 1 for full description of all 68-spacerssubtypes [file 1471-2334-11-42-S1.PDF]

Clade	Number of isolates in the Clade	SIT	68-spacers subtype	Subdivision (Num)
EAI	n=66	48	■■■■■■■■■■■■■■■■■■■■■■■■■■■■■■■■■■■■■■□□□□■□■■■□■■■■■■■■■■■■■□■■■■■■	subtype-1 (n=4)
		48	■■■■■■■■□□■■■■■■■■■■■■■■■■■■■■■■■■■■■■□□□□■□■■■■■■■■■■■■■■■■■□■■■■■■	subtype-2 (n=4)
		48	■■■■■■□■■■■■■■■■■■■■■■■■■■■■■■■■■■■■■■□□□□■□■■■■■■■■■■■■■■■■■□■■■■■■	subtype-3 (n=1)
		48	■■■■■■■■■■■■■■■■■■■■■■■■■■■■■■■■■■■■■■□□□□■□■■■□■■■■■■■■■■■■□□■■■■■■	subtype-4 (n=1)
		204	■■■■■■■■■□□□■■■■■■■■■■■■■■■■■■■■■■■■■■□□□□■□■■■□■■■■■■■■■■■■■■■■■■■■	subtype-1 (n=6)
		204	■■■■■■■■■□□□■■■■■■■■■■■■■■■■■■■■■■■■■■□□□□■□■■■□■■■■■■■■■■□■■■■■■■■■■	subtype-2 (n=1)
		459	■■■■■■■■■■■■■■■■■■■■■■■■■■■■■■■■■■■■■■□□□□■□■□□□□□□■■■■■■■■■■□■■■■■■	subtype-1 (n=5)
		459	■■■■■■■■■■■■■■■■■■■■■■■■■■■■■■■■■■■■■■□□□□■□■□□□□□□■■■■■□■■■■□■■■■■■	subtype-2 (n=1)
		89	■■■□■■■■■□■■■■■■□□□□□□□□□□□□□□□□□□□■■■□□□□■□■■■□■■■■■■■■■■■■■■■■■■■■	subtype-1 (n=5)
		89	■■■□■■■■■□■■■■■■□□□□□□□□□□□□□□□□□□□■■■□□□□■□■■■□■■■■■■■■■□■■■■■■■■■■■■	subtype-2 (n=1)
Beijing	n=30	1	□□ □□□■■■■■■■■□□□□□□□□■■■■■■■	subtype-1 (n=26)
		1	□□□■■■□■■■■□□□□□□□□■■■■■■■	subtype-2 (n=2)
